# Bystander CD4^+^ T cells: crossroads between innate and adaptive immunity

**DOI:** 10.1038/s12276-020-00486-7

**Published:** 2020-08-28

**Authors:** Hong-Gyun Lee, Min-Ji Cho, Je-Min Choi

**Affiliations:** 1 Department of Life Science, College of Natural Sciences, Hanyang University, Seoul, Republic of Korea; 2 Research Institute for Natural Sciences, Hanyang University, Seoul, Republic of Korea; 3 Research Institute for Convergence of Basic Sciences, Hanyang University, Seoul, Republic of Korea

**Keywords:** T cells, CD4-positive T cells

## Abstract

T cells are the central mediators of both humoral and cellular adaptive immune responses. Highly specific receptor-mediated clonal selection and expansion of T cells assure antigen-specific immunity. In addition, encounters with cognate antigens generate immunological memory, the capacity for long-term, antigen-specific immunity against previously encountered pathogens. However, T-cell receptor (TCR)-independent activation, termed “bystander activation”, has also been found. Bystander-activated T cells can respond rapidly and secrete effector cytokines even in the absence of antigen stimulation. Recent studies have rehighlighted the importance of antigen-independent bystander activation of CD4^+^ T cells in infection clearance and autoimmune pathogenesis, suggesting the existence of a distinct innate-like immunological function performed by conventional T cells. In this review, we discuss the inflammatory mediators that activate bystander CD4^+^ T cells and the potential physiological roles of these cells during infection, autoimmunity, and cancer.

## Immunology: bystander helper T cells in health and disease

Immune cells that become activated in the absence of antigen stimulation could be harnessed in the fight against infection, autoimmunity, and cancer. Je-Min Choi and colleagues from Hanyang University in Seoul, South Korea, review how the immune system can deploy helper T cells through an unusual process called bystander activation. Most T cells become activated only after receptors on their surface bind to specific cognate antigen. In contrast, bystander T cells are activated non-specifically in response to cytokines and other pro-inflammatory mediators. Studies have shown that this cell population has a variety of protective and pathogenic functions, for example, guarding against multiple sclerosis, aggravating the symptoms of parasitic infections and promoting antitumor immunity. A better understanding of these immune cells could lead to new therapeutic options for these diseases.

## Introduction

The immune system is classically divided into the innate and adaptive arms^1^. Innate immune cells express invariant antigen receptors, such as pattern recognition receptors (PRRs) that recognize conserved molecular patterns of pathogens. Adaptive immunity is mediated by cells known as lymphocytes, which have evolved to recognize pathogens through highly precise and diverse antigen-specific receptors generated by gene rearrangement. While innate immunity generates less specific but rapid inflammatory responses, adaptive immunity provides long-lasting, highly specific defense and protection against pathogens by generating immunological memory^2^. This dichotomy, supported by the clonal selection theory and the concept of antigen processing and presentation, has been accepted for decades^3,4^. However, the recent discovery of innate lymphoid cells (ILCs) has drawn special attention to the importance of the innate-like function of lymphocytes. ILCs are derived from common lymphoid progenitors, which also give rise to T lymphocytes. ILC subsets, including ILC1, ILC2, and ILC3, mirror the transcriptional and cytokine profiles of the effector CD4^+^ T-helper T_H_1, T_H_2, and T_H_17 subsets^5^. As ILCs are activated by cytokines rather than antigen receptors, this correlation suggests the possibility of T cells having an innate-like capacity. Of note, the concept of antigen-independent “bystander activation” of conventional T cells has been previously reported. An increasing body of evidence suggests that effector/memory T cells can be activated in the absence of antigen stimulation by pro-inflammatory mediators^6^. The antigen-independent activation of bystander effector/memory CD8^+^ T cells has been shown to play an important role in immune responses in viral infection, cancer, and autoimmunity^7–9^. Although the bystander activation of CD8^+^ T cells has been recently reviewed^6,10^, the key concept of T-cell receptor (TCR)-independent CD4^+^ T-cell activation has not been well characterized. In this review, we focus on the understanding of the TCR-independent bystander activation of CD4^+^ T cells and the importance of bystander activation in potential immunological roles and therapeutic approaches during infection, autoimmunity, or cancer.

## Overview of bystander CD4^+^ T-cell activation

**Fig. 1:**
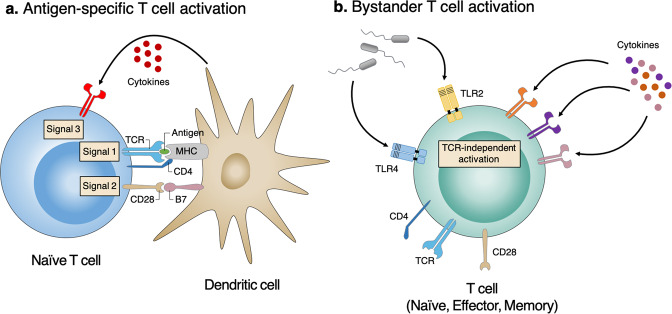
Antigen-specific vs. bystander T-cell activation. **a** Antigen-specific T-cell activation requires three distinct signals. Signal 1 is antigen-specific signaling mediated by T-cell receptor (TCR) engagement of pathogenic peptides presented by major histocompatibility complex (MHC) molecules. Signal 2 is costimulatory signaling, which is mainly mediated by the interaction of CD28 with one of the B7 molecules (CD80 and CD86). Signal 3 is polarizing signaling mediated by various cytokine milieus produced by dendritic cells. **b** In contrast, bystander T-cell activation is the concept of T-cell activation independent of antigen stimulation. Bystander-activated T cells can respond rapidly to inflammatory mediators (cytokine and TLR signaling) in a TCR-independent manner. TLR2 Toll-like receptor 2, TLR4 Toll-like receptor 4.

Traditionally, the activation and differentiation of naïve CD4^+^ T cells into effector T-helper cells require THREE distinct signals: TCR engagement of antigen peptides presented by major histocompatibility complex class II molecules (signal 1) and the interaction of costimulatory molecules (signal 2) initiate T-cell activation. These activated naïve CD4^+^ T cells further differentiate into distinct subsets of helper T cells, including T_H_1, T_H_2, T_H_17, T_reg_, and T_FH_ cells, in different cytokine milieus (signal 3), as defined by their pattern of effector cytokine production and immunological function^11,12^. Compared to conventional T-cell activation, bystander T-cell activation is independent of TCR signaling^6,10^ (Fig. 1). Bystander CD4^+^ T cells were first reported in lymphocytic choriomeningitis virus infection, where TCR-independent proliferation of unrelated CD4^+^ T cells was observed^13^. Bystander proliferation has also been demonstrated with direct injection of lipopolysaccharide (LPS) or with cytokine stimulation^14,15^. Interestingly, the TCR-independent activation of CD4^+^ T cells has primarily been observed in memory (CD44^high^) cells, while naïve (CD44^low^) cells exhibit lower reactivity^13–15^. Thus, it appears that cytokines and innate receptors, such as Toll-like receptors (TLRs), can play important roles in TCR-independent bystander activation and that effector/memory CD4^+^ T cells have a lower threshold than naïve CD4^+^ T cells, which indicates that effector/memory CD4^+^ T cells have a higher probability of undergoing this bystander activation.

## Bystander CD4^+^ T-cell activation: IL-1 family cytokines and STAT activators

**Fig. 2:**
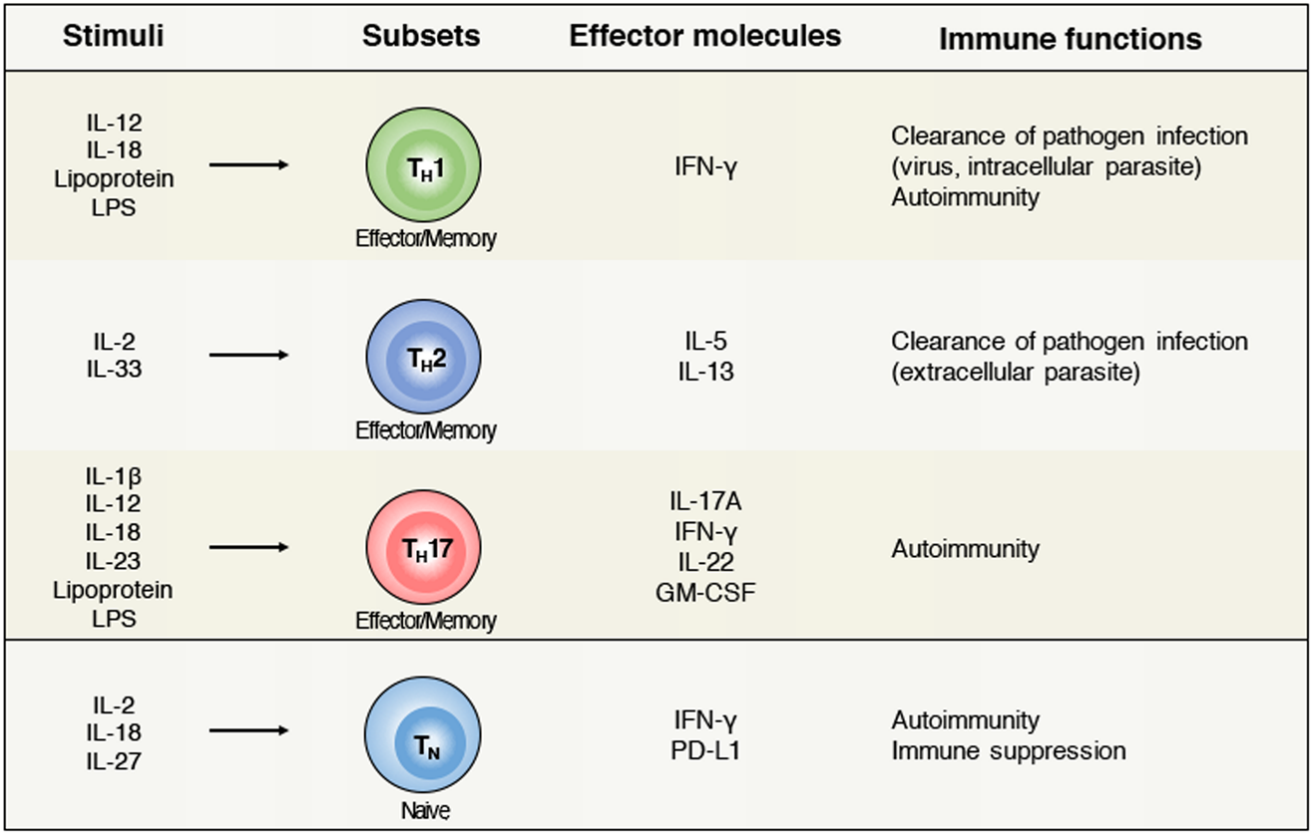
Stimulatory molecules for antigen-independent bystander activation and functioning of CD4^+^ T cells. Effector/memory CD4^+^ T cells (T_H_1, T_H_2, and T_H_17) can undergo bystander activation by directly responding to inflammatory cytokines and TLR agonists. These signals induce effector cytokine production that promotes important pathological responses in autoimmunity and pathogen infections. Naïve CD4^+^ T cells can also be activated in a TCR-independent manner under the influence of cytokines such as IL-2, IL-18, and IL-27. Bystander activation of naïve CD4^+^ T cells can promote immunosuppressive functions that regulate autoimmune pathogenesis. IL interleukin, IFN-γ interferon-γ, GM-CSF granulocyte-macrophage colony-stimulating factor, PD-L1 programmed death-ligand 1, LPS lipopolysaccharide, T_H_1 T-helper 1, T_H_2 T-helper 2, T_H_17 T-helper 17, T_N_ naïve CD4^+^ T cell.

Cytokines are the central mediators that regulate innate and adaptive immune responses. Previous studies have revealed that pro-inflammatory cytokines can directly induce the effector function of T cells^6,10,16^. Of note, Interleukin-1 (IL-1) family cytokines (IL-1, Interleukin-18 (IL-18), and Interleukin-33 (IL-33)) and signal transducer and activator of transcription (STAT) activators (Interleukin-2 (IL-2), Interleukin-12 (IL-12), Interleukin-23 (IL-23), and Interleukin-27 (IL-27)) appear to be potent activators of antigen-independent bystander activation of CD4^+^ T cells (Fig. 2).

### Interleukin-1 (IL-1)

IL-1, the first identified interleukin, is a central mediator of innate and adaptive immune responses^17^. IL-1β has been shown to contribute to the early differentiation and maintenance of T_H_17 cells by regulating the expression of IFN regulatory factor (IRF4) and retinoid acid-related orphan receptor (ROR)γt^18^. Importantly, IL-1β can potently induce cytokine production by effector T_H_17 cells in the absence of TCR engagement^18–20^. As differentiated T_H_17 cells upregulate the expression of IL-23 and IL-12 receptors, IL-1β acts synergistically with IL-23 and IL-12^19,21,22^. In the presence of IL-1β, T_H_17 cells can produce IL-17 and IFN-γ in a TCR-independent manner when stimulated with IL-23^18–21^ or IL-12^21^, respectively. The IL-1β- and IL-23-mediated mechanisms underlying the bystander activation of T_H_17 cells are dependent on nuclear factor (NF)-κB and p38 mitogen-activated protein kinase (MAPK) signaling^20^. We and others have shown that both murine and human memory CD4^+^ T cells express high levels of interleukin receptor type 1 (IL-1R1)^23,24^. Memory but not naïve CD4^+^ T cells primarily respond to IL-1β in the absence of TCR engagement. This bystander activation synergizes with IL-23 to induce pathogenic T_H_17 signature genes (e.g., Csf2, Il23r, Bhlhe40, Ccr6, and Rorc) while downregulating the expression of nonpathogenic T_H_17 signature genes (e.g., Foxp3, Il10, and IL6st). Murine models of multiple sclerosis (MS) (experimental autoimmune encephalomyelitis, EAE) have revealed the potential pathogenicity of antigen-nonrelated memory-like T_H_17 cells responding to IL-1β and IL-23. The recruitment of bystander T cells into the central nervous system (CNS) and their pathogenic function were found to be directly mediated by IL-1R1 signaling^24^.

### Interleukin-18 (IL-18)

IL-18, a member of the IL-1 cytokine family, is a pleiotropic cytokine potentially capable of inducing bystander activation of CD4^+^ T cells in both mice and humans^17^. The TCR-independent function of IL-18 has been shown in various T-helper subsets including T_H_1, T_H_2, and T_H_17 cells^19,21,25^. IL-18 overexpression in the murine lungs spontaneously induced effector CD4^+^ T cells expressing IFN-γ, IL-13, and IL-17A without exogenous antigen challenge^26^. Effector T_H_1 cells extensively express IL-18 receptor α (IL-18Rα), and signaling through this receptor increases the expression levels of IFN-γ and IL-18Rα in direct response to IL-18^19^. In addition, IL-18 promotes IL-17A production in T_H_17 cells generated in vitro or in vivo in the absence of TCR engagement^21^. IL-18 has also been demonstrated to contribute to the bystander activation of both murine and human naïve and memory CD4^+^ T cells to produce high levels of IFN-γ^27,28^. Recent studies have identified a major subset of human mucosal memory CD4^+^ T cells expressing IL-18Rα. IL-18Rα^+^ memory CD4^+^ T cells respond to IL-18 by producing IFN-γ, TNF-α, IL-6, IL-5, IL-13, GM-CSF, and IL-22 in an antigen-independent manner^29^. IL-18 has been shown to have synergistic effects with various combinations of cytokines, including IL-2, IL-12, IL-23, and IL-15. Of note, these “cytokine synergies” were mostly dependent on IL-18 signaling^29^, thus implicating IL-18 as an important initial mediator of the bystander activation of antigen-nonrelated CD4^+^ T cells during host defense.

### Interleukin-33 (IL-33)

IL-33, an epithelial-derived cytokine in the IL-1 family, is a particularly potent activator of ILC2s that induces the production of the effector cytokines IL-5 and IL-13^17,30^. T_H_2 cells, which selectively express the IL-33 receptor (ST2), are another important effector cell type responsive to IL-33. Previous studies have reported that ST2 is expressed on T_H_2 cells but not on T_H_1, T_H_17, or T_reg_ cells^19,31,32^. Because of this ST2 expression pattern, T_H_2 cells are considered to directly respond to IL-33^19,33,34^. In vitro studies have revealed that T_H_2 cells can produce IL-13 and IL-5 but not IL-4 in response to IL-33 without TCR engagement. The antigen-independent activation of T_H_2 cells responding to IL-33 was dependent on NFκB and p38 but independent of nuclear factor of activated T cells^19^. Notably, memory T_H_2 cells express ST2 at higher levels than effector T_H_2 cells^35,36^. A murine infection model demonstrated the innate immunological function of bystander memory T_H_2 cells in allergic inflammation and protection against early helminth infection. This bystander activation of airway memory T_H_2 cells was dependent on IL-33 but not on TCR stimulation^33^. Furthermore, lung-resident ST2^+^ memory CD4^+^ T cells have been reported to be involved in IL-33-induced lung inflammation. Compared with ILC2s, ST2^+^ memory CD4^+^ T cells were the major contributors to the pathogenicity of eosinophilic inflammation and functioned by producing IL-5 and IL-13^34^. Thus, collectively, these results suggested a distinct innate mechanism of bystander-activated effector/memory T_H_2 cells in type-2 inflammation that correlates with ILC2s.

### Interleukin-2 (IL-2)

IL-2, originally called T-cell growth factor, is a member of the common γ-chain family^37^. IL-2 induces STAT5 signaling, which has important roles in the regulation of T-cell proliferation and immune responses^38^. Both in vitro and in vivo studies have revealed that IL-2 drives the expansion and proliferation of bystander CD4^+^ T cells^39,40^. However, the direct effect of IL-2 on bystander-activated CD4^+^ T cells is unknown. When naïve murine CD4^+^ T cells are treated with high doses of IL-2, they can respond to IL-12 and IL-18 to produce IFN-γ in the absence of TCR engagement^27^. This result suggests that high-dose IL-2 stimulation can replace TCR signaling to activate naïve CD4^+^ T cells. Furthermore, IL-2-mediated STAT5 signaling has been shown to be important in the TCR-independent activation of T_H_2 cells. IL-2 is directly involved in GATA3 and ST2 upregulation in T_H_2 cells, which has a synergistic effect with IL-33 to drive the production of the effector cytokine IL-13^19^. Further studies may be necessary to establish the potential roles of IL-2 in the effector functions of bystander-activated CD4^+^ T cells.

### Interleukin-12 (IL-12)

IL-12 is an important cytokine that induces IFN-γ production and T_H_1 differentiation from naïve CD4^+^ T cells through STAT4 signaling. Previous studies have shown that IL-12 is one of the key factors in the induction of TCR-independent IFN-γ production by CD4^+^ T cells in both mice and humans^27,28^. Without antigen stimulation, IL-12 can directly upregulate T-bet and IL-18Rα expression in T_H_1 cells^19^. Together with IL-18, IL-12 induces bystander activation of effector T_H_1 and T_H_17 cells to induce IFN-γ production^19,21^. Studies of in vivo mouse models indicate that IL-12 signaling generates and maintains T-bet^high^ memory-phenotype (MP) CD4^+^ T cells. These MP cells rapidly produce IFN-γ in response to IL-12, which provides host resistance against *Toxoplasma gondii* infection in the absence of cognate antigen recognition^41^. Thus, IL-12 appears to be an important inflammatory mediator of bystander T-cell responses in type-1 immunity.

### Interleukin-23 (IL-23)

IL-23, a pro-inflammatory cytokine belonging to the IL-12 family, is essential for the generation of pathogenic T_H_17 cells^42–45^. IL-23 upregulates the expression of transcription factors, such as RORγt and T-bet, and induces effector molecules, including IL-17A, IFN-γ, GM-CSF, and IL-22, by activating the JAK2/STAT3 signaling pathway^43–47^. We and others have shown that IL-23 alone cannot induce TCR-independent activation of effector/memory CD4^+^ T cells^19,21,24^. However, in the presence of IL-1β, IL-23 increases the expression and production of the effector cytokines IL-17A, IFN-γ, GM-CSF, and IL-22. IL-1β appears to be an important factor in the initial upregulation of IL-23R expression, and compared with either of these two cytokines alone, cotreatment with IL-1β and IL-23 significantly increases the expression levels of both IL-1R1 and IL-23R, indicating the existence of positive feedback between IL-1 and IL-23^24^. Further studies are necessary to reveal the distinct mechanisms by which IL-1β and IL-23 transduce antigen-independent signals to effector/memory CD4^+^ T cells.

### Interleukin-27 (IL-27)

IL-27 is a member of the IL-12 family of heterodimeric cytokines that have immunosuppressive functions. IL-27 can inhibit pro-inflammatory cytokine production by effector T cells, including T_H_1, T_H_2, and T_H_17 cells^48^. Of note, naïve CD4^+^ T cells are reported to express IL-27R, whose expression is downregulated in the presence of TCR signaling. IL-27 can directly upregulate the expression of CD274, which encodes PD-L1, an immune checkpoint inhibitor that binds to PD-1, on naïve CD4^+^ T cells in a STAT1-dependent manner^49^. IL-27-primed naïve T cells inhibit T_H_17 differentiation by expressing PD-L1, which ameliorates the development of autoimmune encephalomyelitis^50^. Hence, IL-27 can induce bystander activation of naïve CD4^+^ T cells, leading to acquisition of antigen-independent inhibitory function. This suggests a potent role for the antigen-independent regulatory function of T cells during inflammation, which merits further investigation.

## Induction of bystander CD4^+^ T-cell activation through TLR signaling

TLRs are one of the key participants in innate immunity. The discovery and functional characterization of TLRs furthered our understanding of how innate immune cells rapidly respond to microbial pathogen invasion^51^. Recent studies, however, demonstrate that T cells can express a variety of TLRs^52^. Direct activation of TLR signaling in effector/memory CD4^+^ T cells induces innate-like effector function (Fig. 2).

### TLR2

TLR2 is an important PRR for the recognition of bacterial lipoteichoic acid, lipoproteins, and peptidoglycans^53^. Previous studies have demonstrated the functional role of TLR2 in effector/memory CD4^+^ T cells^54–62^. TLR2 agonists can act directly on effector T_H_1 cells to induce production of IFN-γ in the absence of TCR stimulation. This TLR2-induced IFN-γ production can be greatly enhanced by the combination of the cytokines IL-2 and IL-12, which induce the activation of MAPK signaling^58^. In addition, T_H_17 cells have been linked to the expression of TLRs, including TLR2 and TLR4. TLR2 signaling, which has a synergistic effect with IL-23 signaling, directly induces the TCR-independent activation and proliferation of T_H_17 cells, resulting in the secretion of the effector cytokines IL-17A and IL-22. A murine model of EAE revealed that T_H_17 cell-intrinsic TLR2 activation is important for the pathogenesis of autoimmune neuroinflammation^57^. ΤLR2 stimulation has been shown to impair the suppressive function of regulatory T cells^61,63,64^. Although TLR2 signaling drives T_reg_ cell glycolysis and proliferation, it reduces the suppressive capacity in an mTORC1-dependent manner. Thus, these findings demonstrate the potential roles of TLRs in regulating T_reg_ metabolism to balance proliferation and suppressive function^61^. In humans, TLR2 is expressed in memory and activated CD4^+^ T cells^54^. In the absence of antigen stimulation, human memory CD4^+^ T cells have been reported to respond to TLR2 agonists with higher IFN-γ production than that achieved by naïve CD4^+^ T cells^54,62^.

### TLR4

TLR4 is a receptor that recognizes for gram-negative bacterial LPS. LPS stimulation has been reported to enhance the proliferation and survival of CD4^+^ T cells. Loss of TLR4 in CD4^+^ T cells abrogated disease symptoms in a murine model of EAE by substantially reducing T_H_17 and T_H_1 cell numbers in the CNS. Thus, TLR4 expression in CD4^+^ T cells is essential in EAE pathogenesis^65^. In contrast, TLR4 signaling in CD4^+^ T cells seems to ameliorate spontaneous colitis. A T-cell transfer model of colitis showed that LPS stimulation inhibited ERK1/2 activation and T_H_1 responses^66^. Regulatory T cells also selectively express TLR4. LPS treatment directly increases the survival/proliferation and suppressor efficiency of T_reg_ cells in the absence of TCR engagement^67^. In human CD4^+^ T cells, LPS stimulation directly increases adherence to fibronectin in a PKC- and p38-dependent manner, indicating that TLR4 signaling can regulate T-cell behavior in inflammation^68^. Thus, TLR signaling is important not only for phagocytic cell functions and inflammation but also direct stimulation of effector T cells, which induces activation of antigen-nonrelated T cells during inflammatory responses.

## Bystander CD4^+^ T-cell activation in immunity and disease

### Infection

The published mechanistic studies on infection are mainly focused on antigen-specific T-cell responses^69,70^. However, the importance of bystander CD8^+^ T cells in viral infections has been recently reviewed^10^, indicating an unexpected role for antigen-nonrelated T cells during infection. Notably, previous studies have revealed the potential role of antigen-nonrelated CD4^+^ T cells in various infectious diseases^33,41,59,71–74^. Bystander activation of CD4^+^ T cells has been reported during herpes simplex virus (HSV) infection. Ocular infection with HSV can cause an immunopathological disease called herpetic stromal keratitis (HSK) in the corneal stroma^75^. HSV-unrelated CD4^+^ T cells were shown to contribute to establishing ocular lesions in the absence of antigen specificity^71,72^, thus indicating a significant role for virus-unrelated CD4^+^ T cells in HSK pathogenesis. Moreover, recent studies have shown evidence for bystander activation of CD4^+^ T cells in parasitic infections^33,41,59^. Mouse models of *Borrelia burgdorferi* infection have demonstrated expansion and activation of *Borrelia*-unrelated TLR2^+^CD4^+^ T cells in the synovial joint. TLR2 expression on bystander T cells contributed to IFN-γ production and arthritis pathogenesis^59^, indicating the existence of a microbe-induced innate mechanism during infection. Recently, T_H_2 cells generated in response to the helminth *Ascaris suum* were shown to contribute to the clearance of unrelated *Nippostrongylus brasiliensis*. OVA-induced airway T_H_2 cells also were found to participate in antigen-nonspecific protection against helminth infection. Such bystander activation of T_H_2 cells was dependent on IL-33 but not on TCR^33^. Furthermore, in *Toxoplasma gondii* infection, compared with pathogen-specific effector T cells, MP CD4^+^ T cells induced rapid IFN-γ production. MP CD4^+^ T cells provided nonspecific IL-12-dependent host resistance against infection^41^. Taken together, these findings indicate that antigen-unrelated CD4^+^ T cells can undergo bystander activation in the absence of TCR stimulation during infection, which could be implicated in both protective and pathogenic immune responses.

### Autoimmunity

**Fig. 3:**
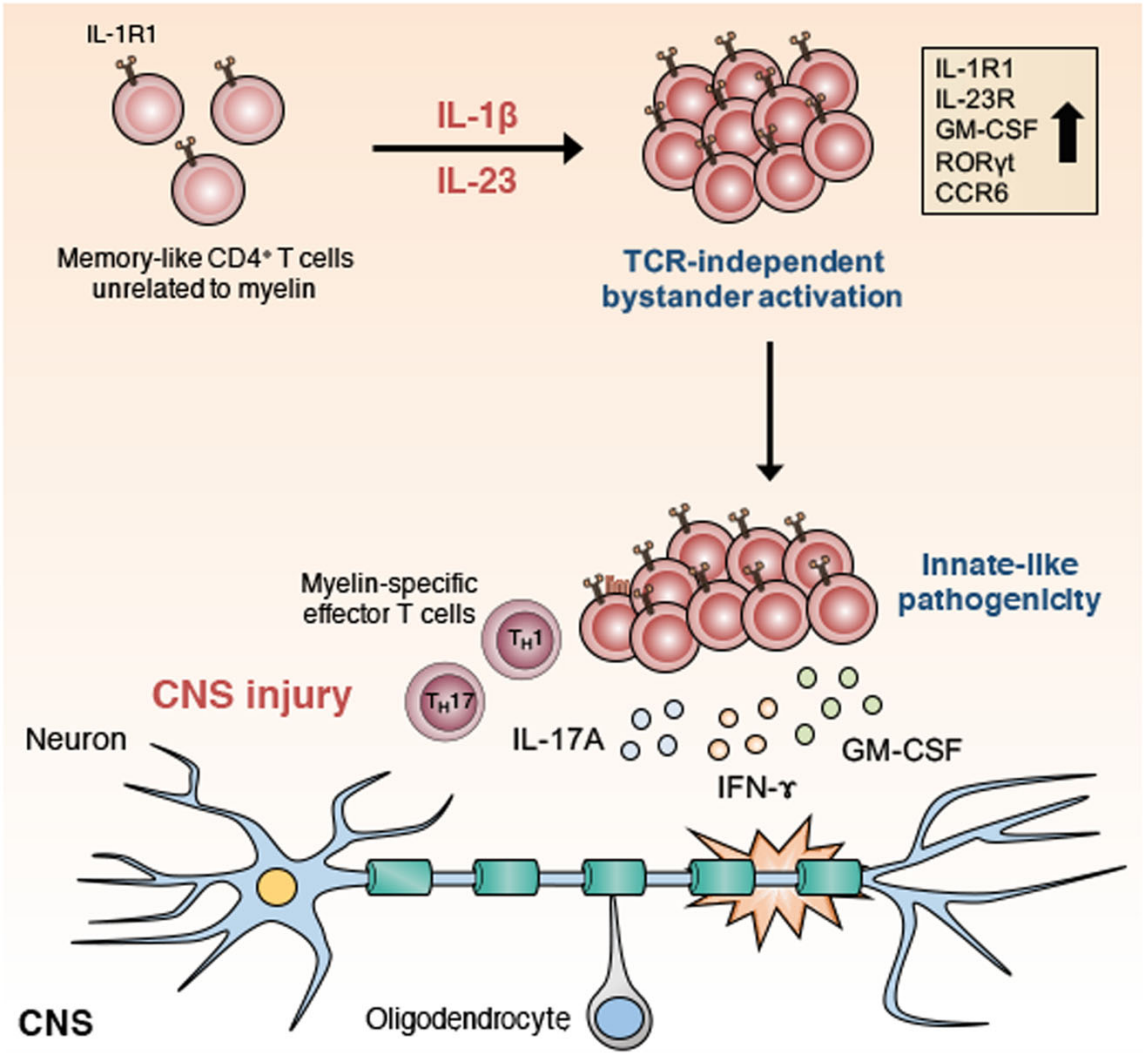
IL-1β and IL-23 induce pathogenic function in bystander-activated memory-like CD4^+^ T cells in autoimmune neuroinflammation. IL-1β and IL-23 induce innate-like pathogenic function in memory-like CD4^+^ T cells that are not specific for myelin. Along with myelin-specific effector T cells (T_H_1 and T_H_17), bystander memory-like CD4^+^ T cells contribute to the development of autoimmune pathogenesis by increasing IL-17A, IFN-γ, and GM-CSF levels in the CNS. Thus, bystander-activated memory-like CD4^+^ T cells responding to IL-1β and IL-23 perform a pathogenic role in an antigen-independent manner in autoimmune encephalomyelitis. IL interleukin, IFN-γ interferon-γ, GM-CSF granulocyte-macrophage colony-stimulation factor, IL-1R1 interleukin-1 receptor type 1, IL-23R interleukin-23 receptor, RORγt RAR-related orphan receptor gamma, CCR6 chemokine receptor 6, T_H_1 T-helper 1, T_H_17 T-helper 17, CNS central nervous system.

MS, one of the most common autoimmune diseases, is an inflammatory demyelinating human autoimmune disease of the CNS^76^. Previous studies on EAE, a model of MS, have reported that myelin-specific T_H_1 and T_H_17 cells mediate the pathogenesis of autoimmune neuroinflammation^77–79^. However, we and other groups have shown that the majority of CNS-infiltrating effector CD4^+^ T cells in the spinal cord of EAE mice are not specific for myelin oligodendrocyte glycoprotein (MOG)^24,80–82^. These T cells unrelated to MOG expressed high levels of effector cytokines (IL-17A, IFN-γ, and GM-CSF)^24^. Murine models of EAE have suggested that effector/memory CD4^+^ T cells can invade the CNS without antigen specificity^24,83–86^. One study reported that T cells unrelated to myelin contributed to EAE development by stimulating the function of antigen-presenting cells^85^. Furthermore, we recently confirmed that memory-like CD4^+^ T cells unrelated to myelin contribute to EAE pathogenicity by amplifying the production of the effector cytokines IL-17A, IFN-γ, and GM-CSF^24^ (Fig. 3), thereby indicating a potential role for bystander CD4^+^ T cells in the pathogenesis of EAE development. GM-CSF, which is directly regulated by the transcription factor RORγt^87^, is a well-defined pathogenic cytokine in T_H_17-related diseases, including EAE^45,87^ and MS^88^. Interestingly, overexpression of GM-CSF in CD4^+^ T cells has been reported to induce spontaneous neuroinflammation regardless of antigen specificity. The transcription factor Bhlhe40, whose expression is a characteristic of encephalitic T_H_17 cells, was primarily expressed in CNS-infiltrating effector T cells unrelated to MOG^82,89^. As further studies have revealed that IL-1β is one of the key cytokines that directly induces GM-CSF and Bhlhe40 expression^45,82,90^, Bhlhe40 and GM-CSF may be potent pathogenic mediators of the bystander activation of CD4^+^ T cells responding to IL-1β. In humans, the CD4^+^ T cells of patients with MS have been reported to significantly express the cytokine receptor IL-1R1 and TLRs, including TLR2 and TLR4, indicating the potential of CD4^+^ T cells to exert innate-like function in MS^55,91^. TLR agonists directly induced IL-6, IFN-γ, IL-17, and GM-CSF production by CD4^+^ T cells in patients with MS^55^. Several groups have also recently reported the protective role of bystander CD4^+^ T cells in the pathology of CNS inflammation^50,92^. T-cell-mediated neuroprotection after CNS injury has been reported to occur in the absence of TCR engagement. This neuroprotective bystander T-cell response was dependent on IL-4, which attenuated axonal damage^92^. In a study of an EAE murine model, naïve CD4^+^ T cells primed with IL-27 induced PD-L1, which inhibited severe autoimmune encephalomyelitis^50^. Together, these findings suggest that bystander CD4^+^ T cells can contribute to the development of and protection against autoimmune encephalomyelitis. Further work is necessary to reveal the precise pathological mechanisms involving bystander CD4^+^ T cells in various autoimmune diseases.

### Cancer

The role of bystander T cells in anticancer immunity remains an open question. However, a recent study revealed that in human lung and colorectal cancers, tumor-infiltrating CD8^+^ TILs can be antigen nonspecific. These bystander CD8^+^ TILs, which lacked expression of CD39, were specific for unrelated viruses including Epstein–Barr virus, human cytomegalovirus, and influenza virus. The absence of CD39 expression, which indicates chronic antigen stimulation, suggested a potent biomarker for bystander T cells in antitumor immunity^8^. Interestingly, murine models of cancer have revealed that CD4^+^ T cells can also infiltrate tumors independent of antigen specificity. TCR-transgenic murine models have demonstrated nonspecific T-cell migration and accumulation in tumors. This bystander activation of CD4^+^ T cells required an effector/memory phenotype^93^. In mice treated with dual costimulation of CD134 (OX40) and CD137 (4-1BB), bystander activation of effector CD4^+^ T cells unrelated to the tumor contributed to the antitumor response, although the precise mechanism is unknown^94^. Collectively, these findings demonstrate the potent role of bystander CD4^+^ T cells in antitumor immunity and immunotherapy. Further investigation of the distinct mechanism involving bystander T cells in tumor pathogenesis may provide a new platform for understanding cancer immunopathology.

## Concluding remarks

Bystander activation of CD4^+^ T cells occurs when T cells are stimulated by inflammatory mediators such as cytokines or TLR signaling molecules in the absence of antigen-specific TCR stimulation. In addition to providing rapid effector cytokine production, these bystander-activated CD4^+^ T cells significantly contribute to disease pathology, including that of infection, autoimmunity, and cancer, via their innate-like capacity. In reality, it may be more than just the few antigen-specific T cells that are capable of responding to pathogen invasion or related inflammation. Therefore, the dichotomy of rapidly responding innate immune cells of low specificity and highly antigen-specific responsive immune memory cells may need to be reconsidered. Concerning the importance of antigen-independent bystander T-cell activation, numerous questions remain unanswered. Further studies dissecting the molecular mechanisms driving bystander T-cell activation and the precise roles of these cells in immunity will provide new insights to improve the understanding of the pathological mechanisms of immune-mediated diseases. In addition, the characterization of specific T-cell subsets undergoing bystander activation needs to be completed. Recently, tissue-resident memory T cells have emerged as key components of immunological memory^95^. They are the dominant T-cell subsets residing in human tissues (the skin, lungs, gastrointestinal tract, etc.) that form a front-line defense against reinfection^96^. Being situated within peripheral tissues, T_RM_ cells may be ideal subset candidates capable of responding to local inflammatory cytokines. Furthermore, identifying distinct and reproducible biomarkers of bystander T cells is necessary for generating new therapeutic approaches. Taken together, these studies will provide a foundation for a better understanding of the pivotal roles of bystander T-cell activation, thereby suggesting new directions for the treatment of immune-mediated diseases.
