# Author Correction: Bystander CD4^+^ T cells: crossroads between innate and adaptive immunity

**DOI:** 10.1038/s12276-023-01032-x

**Published:** 2023-06-08

**Authors:** Hong-Gyun Lee, Min-Ji Cho, Je-Min Choi

**Affiliations:** 1 Department of Life Science, College of Natural Sciences, Hanyang University, Seoul, Republic of Korea; 2 Research Institute for Natural Sciences, Hanyang University, Seoul, Republic of Korea; 3 Research Institute for Convergence of Basic Sciences, Hanyang University, Seoul, Republic of Korea

**Keywords:** T cells, CD4-positive T cells

Correction to: *Experimental & Molecular Medicine* (2020) **52**:1255-1263 10.1038/s12276-020-00486-7, published online 28 August 2020

After online publication of this article, the authors noticed an error in the article the author’s name Min-Ji Cho was incorrectly written as Min-Zi Cho in title page section.

The correct statement of this article should have read as below.

“Hong-Gyun Lee, Min-Ji Cho & Je-Min Choi”

The authors apologize for any inconvenience caused.

The original article has been corrected.

